# Attention-Deficit/Hyperactivity Disorder Symptom Dimensions Differentially Predict Adolescent Peer Problems: Findings From Two Longitudinal Studies

**DOI:** 10.3389/fpsyg.2020.609789

**Published:** 2021-01-13

**Authors:** Shaikh I. Ahmad, Jocelyn I. Meza, Maj-Britt Posserud, Erlend J. Brevik, Stephen P. Hinshaw, Astri J. Lundervold

**Affiliations:** ^1^Department of Psychiatry and Behavioral Sciences, University of California, San Francisco, San Francisco, CA, United States; ^2^Department of Psychiatry and Biobehavioral Sciences, University of California, Los Angeles, Los Angeles, CA, United States; ^3^Division of Psychiatry, Department of Child and Adolescent Psychiatry, Haukeland University Hospital, Bergen, Norway; ^4^Department of Clinical Medicine, Faculty of Medicine, University of Bergen, Bergen, Norway; ^5^Gillberg Neuropsychiatry Centre, University of Gothenburg, Gothenburg, Sweden; ^6^Division of Psychiatry, Haukeland University Hospital, Bergen, Norway; ^7^Department of Biological and Medical Psychology, University of Bergen, Bergen, Norway; ^8^Department of Psychology, University of California, Berkeley, Berkeley, CA, United States

**Keywords:** attention-deficit/hyperactivity disorder, inattention, hyperactivity, impulsivity, peers, peer relationships, cross-cultural research

## Abstract

**Introduction**: Previous findings that inattention (IA) and hyperactive/impulsive (HI) symptoms predict later peer problems have been mixed. Utilizing two culturally diverse samples with shared methodologies, we assessed the predictive power of dimensionally measured childhood IA and HI symptoms regarding adolescent peer relationships.

**Methods**: A US-based, clinical sample of 228 girls with and without childhood diagnosed attention-deficit/hyperactivity disorder (ADHD; *M* age = 9.5) was assessed and followed 5 years later. A Norwegian, population-based sample of 3,467 children (53% girls; *M* age = 8.3) was assessed and followed approximately 4 years later. Both investigations used parent and teacher reports of ADHD symptoms and peer relations. Multivariate regression analyses examined the independent contributions of IA and HI symptoms to later peer problems, adjusting for baseline childhood peer problems. We also examined childhood sex as a potential moderator within the Norwegian sample.

**Results**: Higher levels of childhood HI symptoms, but not IA symptoms, independently predicted adolescent peer problems in the all-female clinical sample. Conversely, higher levels of IA symptoms, but not HI symptoms, independently predicted preadolescent peer problems in the mixed-sex population sample. Results did not differ between informants (parent vs. teacher). Associations between ADHD symptom dimensions and peer problems within the Norwegian sample were not moderated by child sex.

**Discussion**: Differential associations between childhood hyperactive/impulsive and inattention symptoms and adolescent peer problems were found across two diverse samples using a shared methodology. Potential explanations for different findings in the clinical vs. population samples include symptom severity as well as age, sex, and cultural factors. We discuss implications for future research, including the importance of dimensional measures of ADHD-related symptoms and the need for shared methodologies across clinical and normative samples.

## Introduction

Peer relationships, especially throughout childhood and adolescence, have a significant impact on numerous domains of functioning (Bagwell et al., 1998; Mrug et al., 2012). For example, across development, reciprocal relations are shown between peer rejection and externalizing behavior, as well as behavioral and health-related outcomes (e.g., Parker and Asher, 1987; Laird et al., 2001; Dodge and Pettit, 2003; Prinstein and La Greca, 2004; Ladd, 2006). Furthermore, a large body of research reveals that poor peer relationships in childhood predicts depression during adolescence (see Nolan et al., 2003; La Greca and Harrison, 2005; Prinstein et al., 2005; Roy et al., 2015). Yet open questions exist regarding the impact of peer problems on later functioning in clinical vs. population samples. Clinical studies, by definition, focus on individuals who are on the farthest and most pathological end of dimensional syndromes, whereas non-clinical studies – though potentially covering a wide range of functioning and symptom levels – have typically captured individuals on the lower end. To address this knowledge gap, we investigate the predictive value of the two core symptom dimensions of attention-deficit/hyperactivity disorder (ADHD) – inattention (IA) and hyperactivity/impulsivity (HI) – utilizing both a clinical sample including individuals with an ADHD diagnosis and a large population cohort study.

It is well documented that ADHD is related to poor peer relationships (Bagwell et al., 2001; Hoza, 2007; Mrug et al., 2012), as well as many other domains of functioning (Lee and Hinshaw, 2006; Molina et al., 2009; Barkley, 2014). Clear findings are evident in clinical samples (see Kessler et al., 2006; Spencer et al., 2007; Molina et al., 2009; Hinshaw et al., 2012; Barkley, 2014), but symptoms below clinical thresholds may also have negative effects on peer relationships (Hoza et al., 2005; Malmberg et al., 2011). Such negative effects are found even in youth who no longer have severe symptoms of IA and/or HI (Lee et al., 2008). Studies including population-based samples (Andrade et al., 2009; Andrade and Tannock, 2013, 2014; Paap et al., 2013) have therefore been essential in revealing that symptoms of IA and HI are linked with children’s peer relationship problems in representative groups of youth. Importantly, these latter findings also underscore the importance of separately considering the association of dimensionally measured symptoms of IA and HI when prospectively examining their associations with later peer functioning, especially given the instability of ADHD diagnoses over time (Nigg et al., 2010; Willcutt et al., 2012).

The impact of IA and HI on peer relationships is not uniform (Hodgens et al., 2000; Blachman and Hinshaw, 2002; McQuade and Hoza, 2008; Solanto et al., 2009). The most severe peer problems have been reported among individuals with an ADHD-Combined presentation (ADHD-C; i.e., children who display both high HI and IA symptoms), who commonly engage in behaviors leading to increased aggression and consequent rejection by peers (McQuade and Hoza, 2008; Solanto et al., 2009; see Erhardt and Hinshaw, 1994, for data on the extremely fast accrual of peer rejection associated with impulsive aggression). Other studies have shown that severe HI symptoms in primary school children are more strongly associated with classroom disruption than IA, with resultant negative effects on peer relationships. HI is also more predictive of later violence and antisocial behavior in adolescence and young adulthood than IA, even though HI symptoms most often decrease significantly from childhood through adolescence (Wilens et al., 2002). Overall, HI is more strongly associated than IA with comorbid oppositionality and conduct problems in individuals with ADHD, predicting subsequent peer rejection (see Ahmad and Hinshaw, 2016).

On the other hand, the inattentive presentation of ADHD (ADHD-I) is strongly linked to social isolation and withdrawal (Diamond, 2005; Becker et al., 2013). Indeed, compared to typically developing children and children with a combined ADHD diagnosis (ADHD-C), a withdrawn child with ADHD-I is more likely to be neglected by peers and display lower social knowledge (Hinshaw and Melnick, 1995; Maedgen and Carlson, 2000; de Boo and Prins, 2007; McQuade and Hoza, 2008; Gardner and Gerdes, 2015). Moreover, several community-based studies have documented that children’s ability to pay attention – in school, at home, as well as with peers and in social settings – is critically important for the development of peer relationships. Social rules or nuances may be missed by inattentive children (Farmer et al., 2002; Spira and Fischel, 2005; Daley and Birchwood, 2010), preventing them from learning the basic skills necessary to handle the increasing complexity in academic and occupational life (Polderman et al., 2010; Holmberg and Bölte, 2014; Pingault et al., 2014; Lundervold et al., 2017). In a longitudinal follow-up, Tseng et al. (2014) discovered a transactional pattern, whereby IA fueled peer problems, which subsequently contributed to increase IA. Such findings are particularly important because IA predicts later depression/mood problems (Herman and Ostrander, 2007; Connors et al., 2012; Rajendran et al., 2013).

Sex differences documenting the link between ADHD symptoms and peer problems have not been consistently found. Some case–control studies have identified that girls with ADHD symptoms fare worse in their social functioning than boys with such symptoms (e.g., Carlson et al., 1997), whereas other investigations of children with subclinical attention problems have found no significant sex differences (Rielly et al., 2006; Ragnarsdottir et al., 2018). Still others have found that, although boys had higher levels of ADHD symptoms and lower ratings of prosocial behavior than girls, they were no more likely to be rejected by their peers than girls (Diamantopoulou et al., 2005). Furthermore, factors like sex are known to influence the social consequences of ADHD-related behavior (Bellanti and Bierman, 2000; Tseng et al., 2012). In a review, girls with ADHD were found to have significant problems with multiple aspects of peer relations, with effect sizes varying from small to large (Kok et al., 2016).

Herein we investigate contributions of dimensional measures of childhood IA and HI to adolescent peer problems. Crucially, we included two longitudinal samples, each featuring two waves of data. First, we investigate a clinical US sample – the Berkeley Girls with ADHD Longitudinal Study (BGALS; Hinshaw, 2002), composed exclusively of girls, both with and without carefully diagnosed ADHD. Second, we utilize a large Norwegian population-based sample, the Bergen Child Study (BCS; Heiervang et al., 2007), which includes both boys and girls. Both investigations feature similar measures and benefit from a common analytic method. On the basis of the literature reviewed above, we expected that both childhood HI and IA would independently predict increased peer problems in adolescence for the BGALS sample. Less is known about the effects of HI in a population-based sample. We therefore restricted our expectation regarding adolescent peer problems to the IA dimension in the BCS sample. As fewer studies have utilized multiple informants when assessing peer problems, we analyzed information from both teachers and parents separately to investigate informant differences (see Milledge et al., 2019). Given the mixed findings regarding sex differences in HI and IA symptoms and peer relationships, we perform sex-specific analyses within the population-based BCS sample with no *a priori* hypothesis. To investigate potential cultural differences, a final analysis compares results in the typically developing comparison subsample from the BGALS study with results from girls in the BCS sample. We recognize that the two samples and studies were not pre-designed for direct comparison, but, we believe that core similarities in measures, longitudinal design, and inclusion of peer measures can afford a heuristic kind of “systematic replication” regarding the predictive relations between ADHD dimensions and peer-related outcomes.

## Materials and Methods

### Participants

#### BGALS Sample

Girls aged 6–12 (*M* age = 9.5 years) were recruited from schools, mental health centers, pediatric practices, and direct advertisements to participate in summer research programs in the San Francisco Bay Area from 1997 to 1999 as part of the BGALS program (see Hinshaw, 2002). One hundred and forty girls with ADHD (93 with ADHD combined type, 47 with inattentive type) and 88 age-matched and ethnicity-matched comparison girls without ADHD were selected after extensive diagnostic assessment [Wave 1(W1); Hinshaw, 2002].

After initial screening with parent and teacher rating scales, participants with ADHD were included at W1 if they met full ADHD diagnostic criteria for ADHD-C or ADHD-I on the parent-administered Diagnostic Interview Schedule for Children (DISC-IV; Shaffer et al., 2000). Comparison participants were matched to the ADHD sample on age and ethnicity but could not meet diagnostic criteria for ADHD on parent ratings or structured clinical interview. Exclusion criteria for both groups were intellectual disability, pervasive developmental disorders, psychosis or overt neurological disorder, non-English spoken in the home, and a medical problem prohibiting summer camp participation. W1 participants were diverse, both ethnically (53% White, 27% African American, 11% Latina, and 9% Asian American) and socioeconomically (ranging from parents on public assistance through professionals with advanced degrees).

All participants were invited to participate in a comprehensive, prospective follow-up assessment 5 years later (W2; Hinshaw et al., 2006), involving a full-day clinic-based assessment that prioritized multi-domain, multi-source, and multi-informant data collection. The retention rate at W2 was 92% (209 of the 228). Participants lost to follow-up showed no significant differences on 29 out of 31 comparisons. The two exceptions were that those lost to follow-up had higher rates of single-parent households (53 vs. 28%) and higher baseline teacher-reported internalizing scores. W2 participants ranged from 11.3 to 18.2 years (*M* age = 14.2 years). The average time between the W1 and W2 visit was 4.5 years (*SD* = 0.3).

#### BCS Sample

The Bergen Child Study was launched in 2002, through invitations to all parents and teachers of children attending 2–4 grade (7–9 years old; *M* age = 8.3) in any school in the city of Bergen, Norway, asking them to complete questionnaires assessing child mental health (W1; see Heiervang et al., 2007 for details). The response rate was high, with 97% of teachers (*n* = 9,155 children) and 74% (*n* = 7,007) of parents consenting to participate. A follow-up (W2) was conducted approximately 4 years later, in 2006, when the children attended 5–7 grade (ages 11–13 years). Parents and teachers completed questionnaires similar to those used at W1. In all, 5,196 children participated in W2 (response rate: 55.1%). The non-response bias for mean scores and correlations between responders and non-responders participating in W1 was negligible, although the continuing participants tended to show lower symptom scores as reported by teachers on the strengths and difficulties questionnaire (SDQ) than the non-responders (Stormark et al., 2008). Only participants with information from both waves on the selected measures were included in the present analyses (*N* = 3,467, 53% girls). At W1, 42.2% of participants were in second grade, 33.4% in third grade and 24.4% in fourth grade, with equivalent corresponding percentages for 5–7 grades at W2.

### Measures

*ADHD symptom severity*: In both samples, parents and teachers completed the Swanson, Nolan, and Pelham Rating Scale-4th Edition (SNAP; Swanson, 1992), a widely-used and psychometrically sound dimensionalized checklist of DSM-IV-defined ADHD symptoms. The SNAP uses a four-point metric ranging from 0 (not at all true) to 3 (very much true). This range was kept intact for the BGALS investigation but was altered to a three-level metric in the BCS, ranging from 0 (not at all true) to 2 (very much true). The rationale was to make the response metric consistent with other scales included in this study. All nine parent-reported items for each subscale (HI and IA) were summed separately and then divided by nine, to create a mean symptom severity score for each dimension. The same procedure was followed for teachers. Given the high correlations between parent and teacher reports in the BGALS sample (*r* = 0.785, *p* < 0.001 for IA; *r* = 0.660, *p* < 0.001 for HI), we derived a composite score by taking the mean of the mother and teacher scores. The same was then done for the BCS sample to ensure consistency in methodology. The *M* and *SD* for the composite HI symptoms in the BGALS sample were 1.00 and 0.84, respectively; for IA they were 1.44 and 0.97, respectively. For the BCS sample, the *M* and *SD* were 0.13 and 0.22 for HI, respectively; for IA symptoms, they were 0.18 and 0.25, respectively.

*Wave 1 teacher-reported peer problems*. In the BGALS sample, we obtained teacher ratings of peer problems from the Dishion Social Acceptance Scale (Dishion, 1990). We utilized the peer rejection subscale in order to assess the percentage of classmates, estimated by the teacher, who “disliked and rejected” the child on a five-point scale (range 1–5): (1) almost none, less than 25%; (2) a few, around 25%; (3) about half, 50%; (4) most, around 75%; and (5) nearly all, over 75% (see also Hinshaw et al., 2006). The mean and SD for the peer rejection subscale were 1.71 and 1.18, respectively. This measure has been shown to be moderately correlated with the gold standard of peer sociometric nominations (Dishion, 1990).

Teacher ratings of peer problems in childhood for the BCS sample were obtained from the SDQ (Goodman, 2001). The SDQ is a 25-item questionnaire comprising five scales: hyperactivity, emotional problems, conduct problems, peer problems, and prosocial behavior. The SDQ has been widely used, and found to be a good dimensional measure of child and adolescent mental health (Obel et al., 2004; Goodman and Goodman, 2009). The present study utilized the peer problems scale at W1 (range of 0–10, with a higher score indicating more peer relationship problems), which contains items including “tends to play alone,” “is picked on or bullied by other children,” and “is generally liked by other children,” with relevant items being reverse-scored.

*Wave 2 parent and teacher-reported peer problems*. For the BGALS sample at W2, a parent report of adolescent peer problems was used from a project-derived Social Relationships Questionnaire (SRQ; see Hinshaw et al., 2006). The measure contained 12 items on the adolescent’s relationships and friendships on a four-point scale, ranging from 0 (not true) to 3 (very true), with higher scores indicating greater levels of peer problems. In particular, we utilized a peer conflict subscale, based on a principal components analysis that yielded a six-item factor (*α* = 0.83), which included responses such as “My daughter has difficulty making new friends,” “My daughter always had problems with peer relationships,” and “My daughter is often teased, harassed, or picked on by peers.” All six items were summed and then divided by six in order to create an average peer problems score. The mean and SD for parent-reported peer problems were 0.40 and 0.58, respectively.

Additionally, at W2, each participant’s primary teacher completed the Dishion Social Acceptance Scale. The peer rejection subscale was used as a measure of peer problems at W2; it yielded mean and SDs of 1.47 and 0.96, respectively. The parent-reported W2 peer conflict subscale correlated with the W2 teacher-reported Dishion peer rejection subscale at *r* = 0.426 (*p* < 0.001).

For the BCS, both parent and teacher reports on peer problems were assessed by the “peer problems” scale from the SDQ (range of 0–10). The correlation between mother and teacher reported peer problems at W2 was *r* = 0.447 (*p* < 0.001).

*Covariates*. To account for the effects of other relevant factors, we entered the following parent-provided variables at W1 for the BGALS sample: child age, family household income, maternal education, and child’s race/ethnicity. In addition, we included a combined parent/teacher report of W1 anxiety and depression, utilizing the widely-used Child Behavior Checklist and Teacher Report Form (Achenbach, 1991a,b). Specifically, a composite of both parent and teacher reported *t*-scores from the “anxious/depressed” narrow-band syndrome scale was utilized. Finally, we included medication use at W2 as a covariate in our initial models for the BGALS sample, wherein parents reported (yes/no) whether or not participants were either currently taking or had recently taken stimulant medication. At W2, 46 participants were reported as taking stimulant medication for ADHD. For the BCS sample, covariates were restricted to the child’s age at W1, biological sex, and maternal education in all analyses. Mother’s education was included as a proxy for SES, as previous findings for the BCS sample have documented a strong relation between this variable and symptoms in the inattention/hyperactivity domain (Bøe et al., 2012). When analyzing differences between girls and boys in the BCS sample, biological sex comprised the independent variable.

### Data Analytic Plan

We performed multivariate regression analyses to assess the contribution of childhood (W1) IA and HI to adolescent peer problems (W2), separately for parent-reported vs. teacher-reported peer problems. For each model, all covariates were entered at Step 1. As childhood peer problems are often a strong indicator of adolescent peer problems, we then entered W1 peer problems at Step 2, followed by mean HI severity at Step 3, and mean IA severity at Step 4. We then reversed steps 2, 3, and 4 to identify the independent contributions of IA, HI, and W1 peer problems. A parallel analysis was computed for the BCS sample. Also, within the BCS, for HI and IA we created an interaction term with biological sex, to identify potential differences in outcomes between girls and boys, affording a more direct comparison of findings to the all-female BGALS sample. If the interaction term was significant, we tested simple slopes in order to identify the size and direction of effects for girls vs. boys. Of note, in both the parent-reported and teacher-reported models for the BGALS sample, stimulant medication use was not a significant covariate, and in fact reduced overall fit when included in the model. As it did not affect the pattern of findings reported below, it was removed from the final models.

## Results

Descriptive statistics for both studies are provided in Table 1; intercorrelations for all predictor and outcome variables are provided in Table 2 for the BGALS sample and Table 3 the BCS sample.

**Table 1:** Descriptive statistics for predictor and outcome variables.

	BGALS sample (*n* = 228)			BCS sample (*n* = 3,684)							
				Overall			Girls^a^ (*n* = 1942)		Boys^a^ (*n* = 1742)		
	*M*	*SD*	Range	*M*	*SD*	Range	*M*	*SD*	*M*	*SD*	Cohen’s *d*^b^
**Wave 1 Predictors**											
SNAP IA (Parent)	1.6	1.05	(0–3)	0.2	0.29	(0–2)	0.2	0.25	0.3	0.33	0.3
SNAP IA (Teacher)	1.3	1.00	(0–3)	0.1	0.28	(0–2)	0.1	0.21	0.2	0.33	0.4
SNAP IA (Composite)	1.4	0.97	(0–3)	0.2	0.25	(0–2)	0.1	0.19	0.2	0.29	0.4
SNAP HI (Parent)	1.2	0.96	(0–3)	0.2	0.27	(0–2)	0.1	0.21	0.2	0.31	0.4
SNAP HI (Teacher)	0.8	0.88	(0–3)	0.1	0.25	(0–2)	0.0	0.15	0.2	0.32	0.7
SNAP HI (Composite)	1.0	0.84	(0–3)	0.1	0.22	(0–2)	0.1	0.15	0.2	0.27	0.4
Peer functioning (Teacher)	1.7	1.18	(1–5)	0.6	1.29	(0–10)	0.5	1.17	0.7	1.41	0.2
**Wave 2 Outcomes**											
Peer functioning (Parent)	0.4	0.58	(0–2.8)	0.9	1.52	(0–10)	0.8	1.44	1.0	1.60	0.1
Peer functioning (Teacher)	1.5	0.96	(1–5)	0.7	1.41	(0–10)	0.6	1.21	0.9	1.59	0.1

SNAP, Swanson, Nolan, and Pelham Rating Scale, 4th ed.; IA, inattentive symptoms; HI, hyperactive/impulsive symptoms.

a For BCS data, all means differences between boys and girls were significant at the *p* < 0.001 level.

b Cohen’s *d* compares means between boys and girls in the BCS sample.

**Table 2:** Intercorrelations between predictor and outcome variables – BGALS sample.

		Wave 1							Wave 2	
		1	2	3	4	5	6	7	8	9
1	SNAP IA (Parent)		0.79^***^	0.95^***^	0.77^***^	0.54^***^	0.72^***^	0.46^***^	0.42^***^	0.24^**^
2	SNAP IA (Teacher)			0.94^***^	0.62^***^	0.68^***^	0.71^***^	0.51^***^	0.40^***^	0.22^**^
3	SNAP IA (Composite)				0.74^***^	0.64^***^	0.76^***^	0.51^***^	0.43^***^	0.25^**^
4	SNAP HI (Parent)					0.66^***^	0.92^***^	0.49^***^	0.47^***^	0.40^***^
5	SNAP HI (Teacher)						0.90^***^	0.57^***^	0.47^***^	0.37^***^
6	SNAP HI (Composite)							0.58^***^	0.52^***^	0.43^***^
7	W1 Peer Problems (Teacher)								0.43^***^	0.26^**^
8	W2 Peer Problems (Parent)									0.52^***^
9	W2 Peer Problems (Teacher)									

** *p* < 0.01

*** *p* < 0.001.

SNAP, Swanson, Nolan, and Pelham Rating Scale, 4th ed.; IA, inattentive symptoms; HI, hyperactive/impulsive symptoms.

**Table 3:** Intercorrelations between predictor and outcome variables – BCS sample.

		Wave 1							Wave 2	
		1	2	3	4	5	6	7	8	9
1	SNAP IA (Parent)		0.51^***^	0.88^***^	0.77^***^	0.32^***^	0.55^***^	0.38^***^	0.31^***^	0.28^***^
2	SNAP IA (Teacher)			0.86^***^	0.62^***^	0.62^***^	0.59^***^	0.27^***^	0.26^***^	0.31^***^
3	SNAP IA (Composite)				0.74^***^	0.54^***^	0.66^***^	0.38^***^	0.33^***^	0.34^***^
4	SNAP HI (Parent)					0.45^***^	0.86^***^	0.32^***^	0.24^***^	0.21^***^
5	SNAP HI (Teacher)						0.84^***^	0.18^***^	0.18^***^	0.20^***^
6	SNAP HI (Composite)							0.29^***^	0.25^***^	0.24^***^
7	W1 Peer problems (Teacher)								0.32^***^	0.36^***^
8	W2 Peer problems (Parent)									0.44^***^
9	W2 Peer problems (Teacher)									

^***^p < 0.001.

SNAP, Swanson, Nolan, and Pelham Rating Scale, 4th ed.; IA, inattentive symptoms; HI, hyperactive/impulsive symptoms.

### BGALS Sample

Mothers of the participants reported an average total gross household income between $50,000 and $60,000 per year (incomes reported for 1997–1999) and they had, on average, completed “some college.” At W1, mother-rated SNAP IA symptom severity was higher than that reported by teachers (*t* = 3.12, *p* = 0.002, Cohen’s *d* = 0.3); this pattern was similar to that for SNAP HI symptom severity (*t* = 4.63, *p* < 0.001, Cohen’s *d* = 0.4). W1 HI and IA were both significantly correlated with childhood peer problems (*r* = 0.58 and *r* = 0.51 respectively; *p* < 0.001 in both cases).

Table 4 details the results from the multivariate regression analyses. First, for parent-reported peer problems at W2, the overall model was significant, accounting for 34% of the total variance in the outcome variable. W1 HI symptoms independently predicted parent-reported peer problems at W2, accounting for approximately 15% of overall model variance (Δ*R*^2^ = 0.05, *b* = 0.27, *p* < 0.001), whereas W1 IA was not a significant predictor of W2 peer problems (Δ*R*^2^ = 0.00, *b* = −0.05, *p* = 0.42). W1 peer problems independently predicted W2 peer problems but accounted for less overall model variance compared to HI symptoms (Δ*R*^2^ = 0.02, *b* = 0.08, *p* = 0.032).

**Table 4:** Multivariate regressions: predicting adolescent peer problems.

	Overall model			Wave 1 IA^1^			Wave 1 HI^1^			Wave 1 peer problems^1^		
	*F*	*R*^2^	*p*	Δ*R*^2^	B	*p*	Δ*R*^2^	B	*p*	Δ*R*^2^	B	*p*
**BGALS sample outcomes**												
W2 parent reported peer problems^2^	12.45	0.34	<0.001	0.00	−0.05	0.42	0.05	0.27	<0.001	0.02	0.08	0.032
W2 teacher reported peer problems^2^	5.55	0.24	<0.001	0.01	−0.17	0.16	0.05	0.44	0.002	0.01	0.08	0.30
**BCS sample outcomes**												
W2 parent reported peer problems	117.74	0.17	<0.001	0.02	1.33	<0.001	0.00	0.27	0.061	0.05	0.08	<0.001
*Girls only*	48.43	0.12	<0.001	0.02	1.24	<0.001	0.00	0.71	0.008	0.03	0.22	<0.001
*Boys only*	90.03	0.22	<0.001	0.03	1.34	<0.001	0.00	0.09	*ns*	0.07	0.34	<0.001
W2 teacher reported peer problems	136.12	0.19	<0.001	0.03	1.41	<0.001	0.00	−0.05	*ns*	0.07	0.30	<0.001
*Girls only*	52.56	0.13	<0.001	0.03	1.48	<0.001	0.00	−0.21	*ns*	0.04	0.21	<0.001
*Boys only*	93.74	0.22	<0.001	0.03	1.34	<0.001	0.00	−0.09	*ns*	0.09	0.37	<0.001

IA, inattentive symptoms; HI, hyperactive/impulsive symptoms.

1 Information provided based on adding variable at the last step in the regression model.

2 Covariates included in model: child age, family household income, maternal education, child’s race/ethnicity, and W1 anxious/depressed syndrome scale from the CBCL using a composite of parent and teacher-reported *t*-scores.

A similar pattern emerged for teacher-reported peer problems at W2: the overall model was significant, accounting for approximately 24% of total variance in the outcome variable. Childhood HI symptoms independently predicted peer problems in adolescence, contributing significantly to the overall variance (Δ*R*^2^ = 0.05, *b* = 0.44, *p* = 0.002) whereas childhood IA symptoms did not (Δ*R*^2^ = 0.01, *b* = −0.17, *p* = 0.16). Yet unlike the case above for parent-reported peer problems at W2, W1 teacher-reported scores did not independently predict W2 teacher-reported scores and contributed very little variance to the overall model (Δ*R*^2^ = 0.01, *b* = 0.08, *p* = 0.30).

In summary, for both parent and teacher reports: (a) childhood HI symptoms were a significant, independent predictor of adolescent peer problems, even after adjusting for childhood-reported peer problems; (b) childhood IA symptoms did not independently predict adolescent peer problems over and above what was already accounted for by HI symptoms; and (c) models that utilized parent-reports accounted for more overall model variance regarding adolescent peer problems than the teacher-reported model (34 vs. 24%).

### BCS Sample

A total of 6.4% of the mothers completed only primary school education, 36.1% completed some form of high school (either practical or theoretical), 31.8% received a college degree, and 25.7% completed more than 4 years of university education. At W1, parent-rated SNAP IA symptom severity was significantly higher than that reported by teachers (*M* = 0.21 vs. 0.14, respectively; *t* = 10.70, *p* < 0.001, Cohen’s *d* = 0.4). The same pattern was found for HI symptom severity between parents and teachers (*M* = 0.16 vs. 0.10; *t* = 12.35, *p* < 0.001, Cohen’s *d* = 0.4). For the composite scores, both childhood IA symptoms and HI symptoms were positively correlated with childhood peer problems at W1 (*r* = 0.380 and *r* = 0.294, respectively; *p* < 0.001 in both cases). Of note, for all ratings (including IA, HI, and peer problems), boys had significantly higher scores than girls for both parent and teacher reports (*p* ≤ 0.001 in all cases), with Cohen’s *d* ranging from 0.2 to 0.7. Refer to Table 1 for additional details.

Table 4 details the results from the multivariate regression analyses. We first addressed childhood HI and IA symptoms as predictors of parent-reported peer problems at W2. The model accounted for approximately 17% of overall variance in the outcome variable. Here, W1 IA symptoms independently predicted parent-reported peer problems at W2, accounting for roughly 15% of model variance (Δ*R*^2^ = 0.02, *p* < 0.001); but W1 HI symptoms did not independently predict parent-reported peer problems at W2. Additionally, W1 teacher-reported peer problems were a significant predictor of W2 parent-reported peer problems (Δ*R*^2^ = 0.05, *p* < 0.001).

Regarding teacher-reported peer problems at W2, the model accounted for 19% of overall variance. W1 IA symptoms independently predicted peer problems at W2, accounting for roughly 17% of model variance (Δ*R*^2^ = 0.03, *p* < 0.001), but W1 HI symptoms did not independently predict W2 peer problems (Δ*R*^2^ = 0.00, *p* = *ns*). W1 teacher-reported peer problems was again a significant predictor of W2 peer problems, accounting for a substantial amount of model variance (Δ*R*^2^ = 0.07, *p* < 0.001). In summary, (a) across both informants, childhood IA symptoms independently predicted preadolescent peer problems – even after accounting for childhood reports of peer problems, whereas HI symptoms did not; (b) regarding preadolescent peer problems, more model variance was accounted for when teachers were the informant vs. parent-report. Unlike the BGALS sample, childhood teacher-reported peer problems was a stronger predictor of preadolescent peer problems than were IA symptoms.

We then performed moderation analyses in the BCS sample to assess the association between sex-by-IA and sex-by-HI with respect to preadolescent peer problems. Overall, four interaction terms were created: one each for HI and IA related to both parent-reported preadolescent peer problems and teacher-reported preadolescent peer problems. In all four cases, the interaction terms were not significant, suggesting that sex differences were not significant moderators of associations between ADHD symptoms and later peer problems.

Finally, to more directly compare the results in the population-based BCS sample to the BGALS sample, we performed *post hoc* analyses solely using the comparison girls within the BGALS sample (*N* = 88; i.e., the subsample without an ADHD diagnosis), contrasting findings with those obtained for girls in the BCS sample. Overall baseline HI symptom severity for the BGALS comparison subsample was 0.20 (*SD* = 0.22) compared to a mean of 1.5 (*SD* = 0.67) for the ADHD subsample (Cohen’s *d* = 2.6). For baseline IA symptom severity, the comparison subsample had a mean of 0.35 (*SD* = 0.30) compared to 2.1 (*SD* = 0.50) for the ADHD subsample (Cohen’s *d* = 4.2). For parent-reported adolescent peer problems, the overall model did not attain significance (*F*_7,70_ = 1.82, *p* = 0.108), so that we did not interpret results. A similar pattern existed for teacher-reported peer problems in adolescence, with the overall model not attaining significance (*F*_7,48_ = 0.79, *p* = *ns*), accounting for only 10% of overall variance.

## Discussion

We examined the longitudinal association between dimensionally measured symptoms of childhood inattention, and hyperactive/impulsive symptoms, and preadolescent/adolescent peer problems in both a clinical sample and a population-based sample, using similar (though not identical) measures and analytic methods. The independent association between childhood HI symptoms and adolescent peer problems was significant in the clinical sample (BGALS; after adjusting for IA symptoms and childhood peer problems), whereas the relation between childhood IA symptoms and adolescent peer problems was not significant. The opposite pattern was found with the population-based sample (BCS): IA symptoms independently predicted preadolescent peer problems, after accounting for HI symptoms and childhood peer problems, whereas HI symptoms did not. Childhood peer problems, however, had a stronger association with preadolescent peer problems than did IA symptoms in the population-based sample, across both parent and teacher reports. Regarding sex differences in the population-based Norwegian sample, we found that neither IA nor HI was moderated by biological sex when predicting preadolescent peer problems. When more directly comparing girls without ADHD diagnoses in the BGALS sample to girls in the BCS sample, we found that there was poor model fit, perhaps related to the smaller size of the BGALS subsample (*n* = 88) as well as less variance between participants, which left us with uninterpretable results.

As expected, the overall level of IA and HI symptoms was lower in the population-based sample than in the clinical sample. Still, ADHD-related symptoms of HI and IA were significant predictors of peer problems in adolescence, with differences found between the samples: HI was found to be the most salient predictor in the clinical sample, whereas IA was in the population-based sample. The present findings add to the current literature by suggesting that it is likely that HI symptoms, and not IA symptoms, that are the stronger contributor to subsequent peer problems. The findings from the population sample, which suggests the potential for early symptoms of IA to affect later peer relationships, is substantiated by several community-based studies, which emphasize the impact of inattention on a range of negative indicators of later functioning (Farmer et al., 2002; Spira and Fischel, 2005; Daley and Birchwood, 2010). In a previous study from the BCS, poor vigilance and distractibility were reported to be the inattentive symptoms most strongly associated with academic challenges (Lundervold et al., 2017). Such challenges might contribute to peer problems because of associated factors, such as learning disorders, poor sleep quality, and/or general distress (Polderman et al., 2010; Holmberg and Bölte, 2014; Pingault et al., 2014).

Although our findings may have been influenced by cultural and overall demographic differences between the two samples, different sample characteristics are an important factor for consideration. First, the correlations between HI and IA symptoms were higher in the clinical sample than in the population-based sample, a finding that is not surprising given that the majority of the clinical sample was diagnosed with ADHD (predominantly ADHD-C). Importantly, our findings suggest that higher levels of HI symptoms might confer more risk toward peer problems – potentially given the more overt and disruptive nature of HI symptoms. Additional results support this view, as we found that the importance of HI symptoms affecting later peer problems in the BGALS sample occurred chiefly among girls with ADHD – the majority of whom had an ADHD-C diagnosis – and not the comparison subsample (Hinshaw, 2002). This finding is congruent with previous studies revealing that children with high levels of HI symptoms (and/or those with ADHD-C diagnoses) tend to show considerable peer problems (Erhardt and Hinshaw, 1994; Gaub and Carlson, 1997; McQuade and Hoza, 2008).

We featured peer problems reported by parents and teachers as separate outcome variables to aid with identifying potential differences between informants (and peer-related settings). Clear differences between informants in reporting childhood and adolescent behaviors are well documented (see e.g., De Los Reyes and Kazdin, 2005; Valo and Tannock, 2010), especially when the child has homework problems and externalizing behaviors (Takeda et al., 2016). The BGALS results in the clinical sample were therefore surprising. Although the parent-reported model accounted for more variance than the teacher model with respect to peer problems, the predictive findings overall were highly similar across informants. The inclusion of the population-based sample (BCS) afforded evaluation of sex differences, yet we found that child biological sex did not moderate the effect of HI or IA on later peer problems, in line with the mixed findings from previous studies. Although on average boys exhibited slightly higher levels of HI and IA symptoms compared to girls, there were no significant differences in peer functioning. In addition, in the BCS (population sample), baseline childhood peer problems were a strong predictor of adolescent peer problems for both girls and boys.

Still, some of the current findings were supported by previous studies that examined sex differences and behavioral functioning (Rose and Rudolph, 2006; Becker et al., 2013). Teachers were shown to consistently rate girls as less impaired and exhibiting less problematic behavior in the classroom than boys (Bussing et al., 1998, 2003; Pisecco et al., 2001; Ohan and Visser, 2009). These findings may relate, in part, to an assessment or perception bias, for which both parents and teachers tend to be more aware of a boy’s displays of overt behavior, especially in the classroom.

Peer problems in girls, should, however, not be downplayed. Roy et al. (2015) note that although boys have a higher likelihood of experiencing social impairment, girls show a greater sensitivity to peer problems. This sensitivity may explain the high rate of internalizing problems in adolescent girls in population-based and non-clinical samples (Rucklidge and Tannock, 2001; Modin et al., 2011; Lundervold et al., 2016). Of clinical importance, girls tend to experience stronger barriers to mental health services than boys, with biases at every level from parents/teachers to health personnel. Underestimating their difficulties can predict an overrepresentation of boys referred to clinics, especially during the preadolescent and adolescent years (Graetz et al., 2006). Therefore, further longitudinal studies of sex differences related to child and adolescent psychopathology and peer relations are warranted.

A key strength of the present study is the use of two different longitudinal samples, in different countries, enabling comparisons of results obtained *via* similar assessment methods and the parallel data analytic procedures. There is past precedent for such a two-study approach. It adds a contribution to the extant literature by examining experiences and behaviors in childhood predicting later peer problems, being relevant for both clinical and non-clinical samples.

Still, key limitations are noteworthy. First, the measure of peer problems differed between the two studies. Although both study’s measures tap aspects of conflict within the child’s peer group (dislike, rejection, disagreement, and bullying, for example), measures were not identical. Future studies would benefit by coordinating such multi-site assessments before study onset, to minimize variability when comparing the results. There was also a time difference from W1 to W2 between the studies. Specifically, the age range of participants at W2 for the BCS sample (ages 11–13) was somewhat younger than for the BGALS sample (11–18), and there were approximately 4 years separating W1 from W2 for the BCS sample compared to 4.5 years for the BGALS sample. Moreover, given data limitations, we were unable to explore potential mechanisms related to the development of peer problems. Finally, the BGALS sample included only girls, limiting our knowledge of potential sex differences in this US sample. On the other hand, the lack of precise matching of the two sample and studies with respect to variables like timing of longitudinal intervals, ages, and exact measures can be considered a strength, in terms of a variant of systematic replication (see, for example, Schmidt, 2009). That is, such differences in study design and measurement preclude the unequivocal assertion that the divergent findings relate solely to cultural or clinical vs. population-based sampling strategies, yet we maintain the belief that any attempts at replication are a plus for the fields of clinical and developmental science. Although this was not a pre-planned, multi-site study, we believe that the present findings make a contribution to a research field in which there is a lack of studies focusing on peer relations. Finally, we believe that the results may motivate future multi-site studies.

Overall, previous studies examining the relative effect of HI vs. IA symptoms on peer functioning have diverged in their findings. We sought to account for these differences by taking into consideration both ends of the severity spectrum for dimensionally measured IA and HI symptoms, by including data from two different samples characterized by different levels of symptom severity. In summary, our findings suggest that IA is more associated with peer problems in a population-based sample, and HI is more associated with peer problems in a clinical sample. Thus, the findings highlight the importance of sample characteristics when discussing the predictive value of ADHD-related symptoms. In short, given the significant consequences of discordant peer relationships, and given the discrepancies in results emanating from clinical and population-based samples, we suggest that other investigators utilize data from both types of samples in order to enhance generalizability and inform potential assessment and intervention options.

## Data Availability Statement

The datasets presented in this article are not readily available because for the BCS, the data are not publicly available due to restrictions in the approval given by the Regional Committees for Medical and Health Research Ethics in Norway. The data that support the findings of this study are available on request. For the BGALS, a completely de-identified dataset is not yet available; interested individuals can contact SH, hinshaw@berkeley.edu.

## Ethics Statement

The BCS was approved by the Regional Committee for Medical and Health Research Ethics (REC), Western Norway. The BGALS was approved by the Committee for Protection of Human Subjects, University of California, Berkeley. Written informed consent to participate in this study was provided by the participants’ legal guardian/next of kin.

## Author Contributions

SA: design, statistical analysis, and main responsibility for the manuscript. JM: design, interpretation of results, and drafting of the manuscript. M-BP: data collection, interpretation of results, and drafting of the manuscript. EB: interpretation of results and drafting of the manuscript. SH: data collection, design, interpretation of results, and main comments on the manuscript. AL: data collection, design, statistical analysis, interpretation of results, and drafting of the manuscript. All authors contributed to the article and approved the submitted version.

### Conflict of Interest

The authors declare that the research was conducted in the absence of any commercial or financial relationships that could be construed as a potential conflict of interest.
